# The Association of *GSTT1, GSTM1*, and *TNF-*α Polymorphisms With the Risk and Outcome in Multiple Myeloma

**DOI:** 10.3389/fonc.2019.01056

**Published:** 2019-10-11

**Authors:** Szymon Zmorzyński, Sylwia Popek-Marciniec, Aneta Szudy-Szczyrek, Magdalena Wojcierowska-Litwin, Iwona Korszeń-Pilecka, Sylwia Chocholska, Wojciech Styk, Marek Hus, Agata A. Filip

**Affiliations:** ^1^Department of Cancer Genetics With Cytogenetic Laboratory, Medical University of Lublin, Lublin, Poland; ^2^Chair and Department of Hematooncology and Bone Marrow Transplantation, Medical University of Lublin, Lublin, Poland

**Keywords:** *GSTT1*, *GSTM1*, *TNF-alpha*, rs1800629, rs361525, polymorphism, bortezomib, apoptosis

## Abstract

Oxidative stress and systemic inflammation are closely linked with increased risk of cancer development. Tumor necrosis factor alpha (TNF-α) is one of the pro-inflammatory cytokines. Glutathione S-transferases (GSTs) are enzymes involved in oxidative stress handling. Polymorphisms of genes encoding mentioned molecules may potentially influence the risk and the outcome in neoplastic diseases. Multiple myeloma (MM) is a hematological malignancy characterized by clonal, atypical plasma cell proliferation. In the present study we investigated the association of deletion polymorphisms in *GSTT1/GSTM1* genes and single nucleotide polymorphisms (SNPs) in the *TNF-*α gene at positions −308/−238 with the risk and outcome in MM and sensitivity to bortezomib under *in vitro* conditions. One hundred newly diagnosed MM patients and 100 healthy blood donors were genotyped by means of multiplex PCR (for GSTs) and PCR-RFLP (for TNF-α). In a subgroup of 50 MM patients, bone marrow cells were treated with bortezomib *in vitro*. Patients with −238GA+AA or *GSTT1*-null genotypes had 2.0 (*p* = 0.002) or 2.29 (*p* = 0.013) fold increased risk of MM. The interaction effects and risk of MM were observed in *GSTT1/GSTM1*-null (OR = 2.82, *p* = 0.018), −308/−238GA+AA (OR = 5.63, *p* < 0.001), as well as in all combinations of −308 with GSTs. The −308/−238GA+AA genotypes in comparison to GG were associated with earlier MM onset−61.14 vs. 66.86 years (*p* = 0.009) and 61.72 vs. 66.52 years (*p* = 0.035), respectively. Patients with *GSTM1*-present had shorter progression-free-survival (15.17 vs. 26.81 months, *p* = 0.003) and overall-survival (22.79 vs. 34.81 months, *p* = 0.039) compared with *GSTM1*-null. We did not observe relationship between response rate and studied polymorphisms. The *in vitro* study revealed significantly higher number of apoptotic cells at 12 nM of bortezomib in *GSTT1*-present, *GSTM1*-null/present, −308GG and −238GG/GA+AA genotypes. Our findings comprise large analysis of studied polymorphisms in MM.

## Introduction

Multiple myeloma (MM) is a malignant clonal expansion of plasma cells in the bone marrow (1). Some genetic, biochemical, and environmental factors are involved in the etiology and pathogenesis of this disease (2). DNA damage is one of the crucial causes of MM development and may be caused by free radicals (3, 4).

The reactive oxygen species (ROS) neutralization and cellular oxidative stress handling involves glutathione (GSH) system, which consists, among others, of glutathione-S-transferases (GSTs) (5, 6). GSTs are involved in the elimination of several chemical carcinogens and protect cellular DNA against ROS-induced damage. This enables genomic maintenance (7). The ability to metabolize carcinogens varies between individuals. These differences depend on polymorphisms of genes coding GST theta-1 and GST mu-1 enzymes – *GSTT1* (*locus* 22q11.2) and *GSTM1* (*locus* 1p13.3), respectively (4, 6). The polymorphisms of *GSTT1* and *GSTM1* genes are present in the form of null genotypes, which are caused by deletion of both alleles at a single *locus*. The *GSTT1* and *GSTM1* null polymorphisms are located in coding region. *GSTM1* null polymorphism means that all exons (count = 6) and introns are removed (6 kbp deletion), but promoter and other non-coding regions (5′UTR, 3′UTR) are present. Larger deletion (9 kbp) is observed in *GSTT1* gene (exon count = 8), and it causes loss of structural gene and some regions of flanking sequences. Null genotype results in a complete lack of corresponding enzyme activity (8).

ROS are involved in inflammation development and tumor necrosis factor alpha (TNF-α) secretion (9, 10). TNF-α is a macrophage-derived pro-inflammatory cytokine which may have either an apoptotic or survival activity in MM (11). *TNF-*α gene (*locus* 6p21.33) contains single nucleotide polymorphisms (SNPs) at positions −308 (rs1800629) and −238 (rs361525) in the 5′ promoter region. Both SNPs are characterized by the substitution of guanine (G) by adenine (A). In the case of both −308G>A or −238 G>A polymorphisms the presence of A-allele is associated with higher transcription rate and TNF-α production (12). Enhanced expression of TNF-α correlates with an increased aggressiveness of MM (13).

The introduction of proteasome inhibitors and new immunomodulatory drugs (IMiDs) in the treatment of MM resulted in improvement of overall survival (OS) relative to previous observations (14, 15). Bortezomib, as a proteasome inhibitor, induces an apoptotic cascade, which is preceded by ROS generation (16). Thalidomide can induce a formation of ROS and inhibits TNF-α expression (17). The correlations between response to treatment and studied genotypes have been not thoroughly researched in MM.

In the current study, we investigated the influence of polymorphisms in *GSTT1, GSTM1*, and *TNF-*α genes as genetic indicators of risk and progression of MM. A few similar studies have assessed the significance of *GSTT1* and *GSTM1* polymorphisms in MM (18, 19). However, these reports did not examine the relationship between the efficacy of bortezomib treatment (*in vivo* and *in vitro*) and combination with −308 G>A and −238G>A *TNF*-α polymorphisms. It is very important to define the role of both *TNF*-α polymorphisms in MM.

In this paper we present analysis for studied genetic variants individually and in different combinations to evaluate the role of these genes in modifying the susceptibility toward MM, as well as survival and response to treatment with thalidomide and/or bortezomib. Furthermore, we have analyzed whether these polymorphisms predict sensitivity to bortezomib in cell cultures derived from studied patients. To our knowledge the presented results were not previously published by other authors.

## Patients and Methods

### Patients and Samples

Bone marrow aspirates and peripheral blood samples were collected from 100 newly-diagnosed patients with MM, who were hospitalized at the Chair and Department of Hematooncology and Bone Marrow Transplantation, Medical University of Lublin in years 2013–2019. The study obtained a positive opinion from the Bioethics Committee (no. KE-0254/165/2013 and no. KE-0254/337/2016), according with the ethical standards established by the Helsinki Declaration. The patients and healthy blood donors provided written informed consent.

The characteristics of MM patients is shown in Supplementary Table 1.

Therapeutic induction regimens consisted of thalidomide and/or bortezomib combined with steroids and/or cyclophosphamide. 37 MM patients underwent autologous hematopoietic stem cell transplantation (auto-HSCT). Response to treatment was evaluated using the International Myeloma Working Group guidelines, and classified as stringent complete remission (sCR), complete response (CR), very good partial response (VGPR), partial response (PR), minimal response (MR), stable disease (SD) or progressive disease (PD) as described elsewhere (20, 21). Overall survival (OS) encompassed time from diagnosis until relapse, progression, death due to tumor effect or last follow-up, and time from diagnosis until death by any cause or last follow-up, respectively. The median follow-up time of MM patients enrolled in the study was 18 months. Progression free survival (PFS) was estimated as the time elapsed between treatment initiation and tumor progression or death from any cause (22).

Peripheral blood was used for DNA isolation and to determine polymorphisms. Cell cultures were established from bone marrow aspirates to carry out the research associated with cIg-FISH (*n* = 100) and bortezomib treatment (*n* = 50). MM patients without chromosomal aberrations were included in the *in vitro* bortezomib study.

Control samples were made of peripheral blood obtained from 100 healthy blood donors (50 males and 50 females) attending the Regional Blood Donation and Blood Treatment Center in Kielce. The mean age of blood donors was 34.4 years (range 18–61 years). The inclusion and exclusion criteria for MM patients and control group are shown in Supplementary Table 2.

### DNA Isolation

DNA isolation from peripheral blood was performed using a commercial kit (Qiagen, Germany) according to manufacturer's procedure. The concentration and quality of DNA was checked using the NanoDrop device (Thermo Fisher Scientific, USA).

### Genotyping

**Figure 1:**
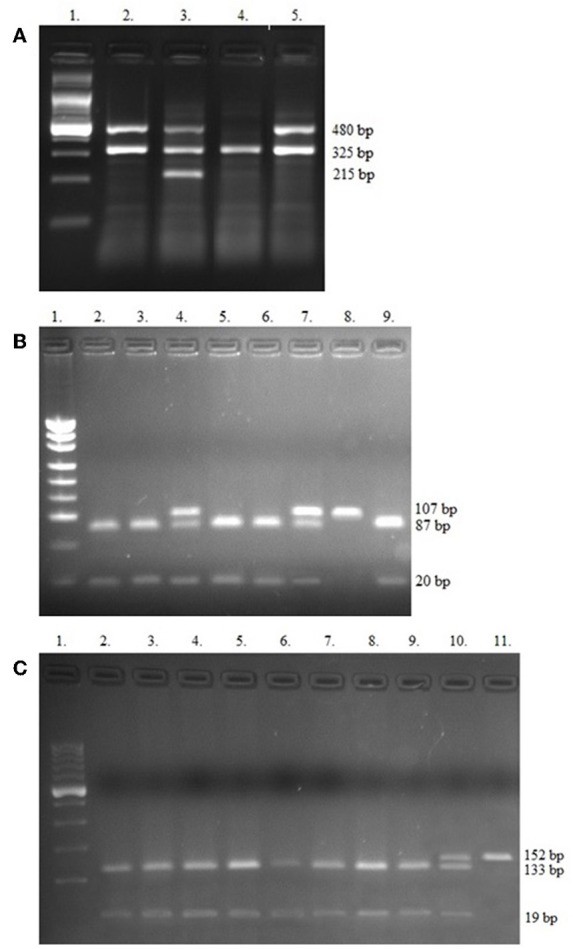
Electropherograms of studied polymorphisms. **(A)** Multiplex PCR of *GSTT1, GSTM1*, and internal control beta-globin genes, Lane 1—Ladder (100 bp); Lanes 2, 3, and 5 contain 480 bp band (*GSTT1*present); Lane 3 shows 215 bp (*GSTM1*present); Lane 4—only 325 bp for internal control (*GSTT1*null/*GSTM1*null); **(B)** PCR-RFLP of −308 *TNF-*α SNPs, Lane 1—Ladder (consists of 34, 67, 110, 147, 190, 242, 331, 404, 489 bp bands); Lanes 2, 3, 5, 6, and 9 contain 87 and 20 bp (GG genotypes); Lanes 4 and 7 show GA genotypes (107, 87, and 20 bp bands), Lane 8 contains 107 bp band (AA genotype); **(C)** PCR-RFLP of −238 *TNF-*α SNPs. Lane 1—Ladder (100 bp), Lanes 2–9 show GG genotypes (133 and 19 bp bands); Lane 10 contains 152, 133, and 19 bp band (GA genotype) and Lane 11 shows one band (152 bp)—AA genotype.

For analysis of *GSTT1* and *GSTM1* polymorphisms, the multiplex PCR method was applied. The −308 (rs1800620) and −238 (rs361525) polymorphisms of *TNF-*α gene were assessed by PCR restriction fragment length polymorphisms (PCR-RFLP). The primers, enzymes used and band sizes obtained in multiplex PCR and PCR-RFLP methods are shown in Table 1. The PCR products were analyzed on 3% agarose gel and stained with SimplySafe (Eurx, Poland) and visualized in G:Box (Syngene, Great Britain) (Figure 1). An independent PCR analysis was carried out for each sample.

**Table 1:** Characteristics of PCR primers and analyzed fragments.

**Polymorphism**	**Primer sequence**	**Method of genotyping**	**Analyzed fragments (bp)**
*GSTT1* null/present	P1F 5′-TTCCTTACTGGTCCTCACATCTC-3′ P1R 5′-TCACCGGATCATGGCCAGCA-3′ P2F 5′-GAAGAGCCAAGGACAGGTAC-3′ P2R 5′-TGGTCTCCTTAAACCTGTCTTG-3′	PCR multiplex with β-globin gene	Null: no band Present: 480 bp Internal control: 325 bp
*GSTM1* null/present	P3F 5′-GAACTCCCTGAAAAGCTAAAGC-3′ P3R 5′-GGTGGGCTCAAATATACGGTGG-3′ P4F 5′-GAAGAGCCAAGGACAGGTAC-3′ P4R 5′-TGGTCTCCTTAAACCTGTCTTG-3′	PCR multiplex with β-globin gene	Null: no band Present: 215 bp Internal control: 325 bp
*TNF-α* −308 G>A	P5F 5′-AGGCAATAGGTTTTGAGGGCCAT-3′ P5R 5′-TCCTCCCTGCTCCGATTCCG-3′	PCR-RFLP with NcoI	G allele: 87 and 20 bp A allele: 107 bp
*TNF-α* −238 G>A	P6F 5′-AGAAGACCCCCCTCGGAACC-3′ P6R 5′-ATCTGGAGGAAGCGGTAGTG-3′	PCR-RFLP with MspI	G allele: 133 and 19 bp A allele: 152 bp

#### *GSTT1* and *GSTM1* Genotyping

For the multiplex PCR each reaction mixture (25 μl) contained 100 ng genomic DNA and PCR buffer (Clontech Laboratories, USA), dNTPs mixture (0,25 mM), HD polymerase (Clontech Laboratories, USA) and primers (10 μM of each). The method used was by Abdel-Rahman et al. with minor modifications (23). The mixture was heated in 94°C for 5 min and underwent 35 cycles of amplification: denaturation 94°C for 2 min, annealing 59°C for 1 min, and elongation 72°C for 1 min. The final elongation took 10 min at 72°C. The PCR reaction was performed in an Applied Biosystems 9700 Thermal Cycler.

#### *TNF*-α Genotyping

The −308 *TNF-*α polymorphism was analyzed according to the Wilson's protocol with modifications (24). PCR mixture (25 μl) contained 100 ng genomic DNA and PCR buffer (ADS Biotec, USA), dNTPs mixture (0.25 mM), Optimase polymerase (ADS Biotec, USA), and primers (10 μM of each). The mixture underwent touchdown PCR including 95°C for 2 min and 7 cycles of amplification: denaturation 95°C for 30 s, annealing 66°C for 30 (−1°C per cycle), elongation 72°C for 20 s; followed by 30 cycles with denaturation 95°C for 30 s, annealing 59°C for 30, elongation 72°C for 20 s. The final elongation took 3 min at 72°C. The PCR reaction was performed in an Eppendorf Mastercycler.

The −238 *TNF-*α polymorphism was characterized by PCR-RFLP (25). Each PCR mixture (25 μl) contained 100 ng genomic DNA and PCR buffer (Clontech Laboratories, USA), dNTPs mixture (0,25 mM), HD polymerase (Clontech Laboratories, USA) and primers (10 μM of each). The mixture was heated in 94°C for 4 min and underwent 35 cycles of amplification: denaturation 98°C for 20 s, annealing 61°C for 20 s, elongation 72°C for 25 s. The final elongation took 3 min at 72°C. The PCR reaction was performed in an Applied Biosystems 9700 Thermal Cycler.

### Cytogenetic Analyses

Abnormalities essential for MM, such as del(17p13.1) and *IgHV* gene rearrangements—*t*(4;14), *t*(14;16) were tested by cIg-FISH according to Ross et al. recommendations (26). Cultured bone marrow malignant plasma cells from 100 patients were identified using simultaneous staining of cytoplasmic immunoglobulin and FISH (cIg-FISH) according to the previously described protocol with modifications (27, 28). The following probes, all from Abbott Molecular (Abbott Park, IL, USA), were used: Vysis TP53/CEP 17 FISH Probe Kit for detection of del(17p13.1), Vysis IGH/FGFR3 DF FISH Probe Kit for detection of *t*(4;14)(p16;q32), and Vysis IGH/MAF DF FISH Probe Kit for detection of *t*(14;16)(q32;q23). Fluorescent microscopic analysis was performed by scoring 100 AMCA-positive plasma cells to determine the frequency of each aberration. Cut off level were 20% for deletion probes and 10% for dual fusion probes, according to the recommendations of the European Myeloma Network (26).

### Cell Cultures, Apoptosis, and Necrosis Detection

**Figure 2:**
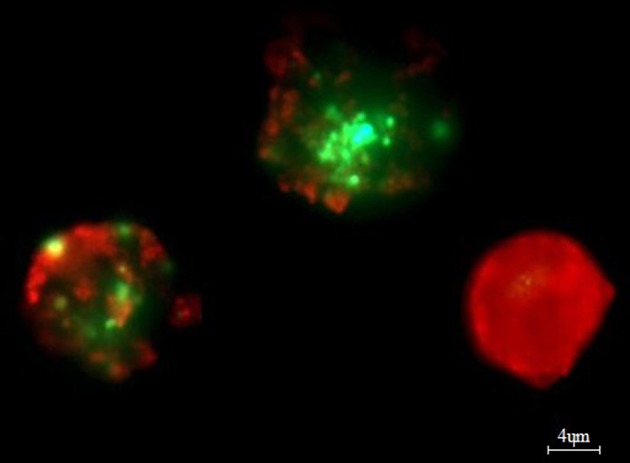
Example of *in vitro* bortezomib activity (at 4 nM). Note two apoptotic cells, which are stained both AnnCy3 (red) and 6-CF (green) and one necrotic cell (red). For analysis plasmocytes with diameter 9–12 μm were counted. Cells were stained by immunofluorescent technique as described in Material and methods. Total magnification of 1.500×.

Bone marrow aspirates (*n* = 50) (mean number of plasma cells was 31.31% ± 20.69) were stratified on Lymphoprep (Axis-Shield PoC As, Norway) and lymphocyte fraction was used to established cell cultures, which were grown in 15 ml of culture medium—RPMI 1640 with L-glutamine (Biomed, Poland); 10% inactivated fetal calf serum (Biomed, Poland), 1% antibiotic antimycotic (A&E Scientific, Belgium), and different doses of bortezomib (LC Laboratories, USA, 200 mg/ml)−1 nM/2 nM/4 nM/8 nM/12 nM. Bortezomib was dissolved in DMSO and stored at −80°C. The final DMSO concentration in culture medium was <0.1%. As a control, cell cultures without bortezomib (with 0.1% DMSO) were used. The amount of 1–1.5 ml of lymphocyte fraction (from each patient) was added, respectively, to the 15 ml of culture medium. The cultures were grown at 37°C in the atmosphere of 5% CO_2_ for 24 h (without granulocyte colony-stimulating factor). The cell cultures were routinely terminated and cell suspensions were prepared to determine the number of apoptotic, necrotic, and viable cells by means of Annexin V-Cy3 Apoptosis Detection Kit according to manufacturer's protocol (Sigma-Aldrich, USA). For fluorescence microscopy, viable cells were stained with 6-CF (6-carboxyfluorescein)—green, necrotic cells were stained only with AnnCy3 (Annexin V Cy3.18). Cells starting apoptotic process were stained both with AnnCy3 (red) and 6-CF (green) (Figure 2). Plasma cells (with diameter 9–12 μm) were analyzed using fluorescence microscope according to Carter's et al. (29).

### Statistical Analysis

Laboratory values of MM patients with polymorphisms were compared using an independent *t*-test for continuous variables and Chi-square test for categorical variables. The association of studied polymorphisms with prognostic factors was evaluated using Chi-square test or Fischer's exact test (when one expected value was <5). The quantitative data was shown as a frequency or percentage. Deviation of genotype frequencies in controls and cases from Hardy-Weinberg equilibrium (HWE) was assessed by Chi-squared test with Yates's correction for the groups with <5 patients (30). For the 95% confidence interval (CI), we assumed *p* = 0.05 and χ^2^ = 3.84; therefore, if the χ^2^ ≤ 3.84 and the corresponding *p* ≥ 0.05 then the population is in HWE. The CubeX software was used to analyze linkage disequilibrium of the studied TNF polymorphisms (31). Logistic regression was used to evaluate the fold risk of MM. The Cox proportional hazard model was used for univariate and multivariate analysis of OS and PFS. Variables of the International Staging System (ISS), auto-HSCT, and genotypes (without combinations) were included in the multivariate analysis. The Kaplan-Meier method and the log-rank test were used for survival analysis. We assumed a 5% error of inference and the related level of significance *p* < 0.05 would point to the existence of statistically significant differences. Statistical analysis was performed using the Statistica ver. 12.5 (StatSoft) software.

## Results

The presented study included 100 MM patients, 53 males and 47 females, with a median age of 65.32 years. Detailed clinical characteristics of the MM patients is shown in Supplementary Table 1. Genotyping was successful in all the individuals investigated within the study. The HWE test confirmed that the allelic frequencies (of *TNF-*α) for healthy individuals (controls) and MM patients were balanced (Table 2). Linkage disequilibrium between −308 G>A and −238 G>A of *TNF-*α polymorphisms were seen in the study (D' = 1, *r*^2^ = 1). The allelic frequencies of both *TNF-*α polymorphisms between study and control groups were statistically insignificant (Table 3). The GA and AA genotypes [of *TNF-*α −308 G>A and −238 G>A polymorphisms] were analyzed together, because the number of AA cases was low. The patients with GA+AA genotypes of *TNF-*α −238 G>A had a 2.0-fold increased risk of MM (*p* = 0.024) compared with GG genotypes (−238 G>A). Similar findings were observed in patients with the *GSTT1* null genotype (OR = 2.29, *p* = 0.013) (Table 4). The effects of *GST* and *TNF* polymorphisms *loci* on the risk of MM were showed in Table 5. Individuals with *GSTT1* null and *GSTM1* null or *TNF-*α −308 GA+AA and −208 GA+AA genotypes showed an increased risk of MM when compared with those with *GSTT1* present and *GSTM1* present or −308 GG and −238 GG genotypes, with OR of 2.82 (*p* = 0.018) and 5.63 (*p* < 0.001), respectively (Table 5). Similar effects were observed in all combinations of *GSTT1, GSTM1*, and −308G>A, and in some combinations of *GSTT1, GSTM1*, and −238G>A (Table 5).

**Table 2:** Hardy-Weinberg equilibrium for *TNF-*α polymorphisms in case and control groups according to expected (E) and observed (O) values.

	**GG**	**GA**	**AA**	**Total**	HWE *p*-value and χ^2^^*^
Control	*TNF-α* −308 G>A				
E	70	27	3	100	*p* = 0.95, χ^2^ = 0.08
O	72	24	4	100	
Case					
E	75	23	2	100	*p* = 0.71, χ^2^ = 0.68
O	73	27	0	100	
Control	*TNF-α* −238 G>A				
E	64	32	4	100	*p* = 0.74, χ^2^ = 0.59
O	60	38	2	100	
Case					
E	73	25	2	100	*p* = 0.87, χ^2^ = 0.27
O	75	23	2	100	

* *if the χ^2^ ≤ 3.84 and the corresponding p ≥ 0.05 then the population is in HWE*.

**Table 3:** The comparison of *TNF-*α allele frequencies among MM patients and controls.

***TNF* gene alleles**	**MM cases (*n* = 100) *n* (%)**	**Controls (*n* = 100) *n* (%)**	***p*-values**
***TNF-α−308 G>A***			
G	172 (86)	168 (84)	0.57
A	28 (14)	32 (16)	
Total:	200 (100)	200 (100)	–
***TNF-α−238 G>A***			
G	172 (86)	158 (79)	0.06
A	28 (14)	42 (21)	
Total:	200 (100)	200 (100)	–

**Table 4:** Comparison of the impact of *GST* and *TNF-*α polymorphisms on the risk of MM.

**Genotypes**	**MM patients *n* = 100**	**Controls *n* = 100**	**OR**	**95%CI**	***p-*value**
***GSTT1***					
Present	83	68	Referent	–	–
Null	17	32	2.29	1.17–4.49	**0.013**
***GSTM1***					
Present	73	60	Referent	–	–
Null	27	40	1.80	0.99–3.27	0.051
***TNFα*** **−308G>A**					
GG	73	72	1	–	
GA+AA	27	28	1.05	(0.56–1.95)	0.874
***TNFα*** **−238G>A**					
GG	75	60	1	–	
GA+AA	25	40	2.0	(1.09–3.65)	**0.024**

*The bold values represent significant values*.

**Table 5:** Combination effect of *GST* and *TNF-*α polymorphisms on the risk of MM.

***GSTT1***	***GSTM1***	***TNF* −308 G>A**	***TNF* −238 G>A**	**MM patients n**	**Controls *n***	**OR**	**95%CI**	***p*-value**
***GSTT1*** **and** ***GSTM1***								
Present	Present	–	–	65	46	R	–	–
Null	Present	–	–	8	14	2.47	0.96–6.37	0.056
Present	Null	–	–	18	22	1.72	0.83–3.57	0.140
Null	Null	–	–	9	18	2.82	1.16–6.84	**0.018**
***TNFα*****−308G>A and−238G>A**								
–	–	GG	GG	61	30	R	–	**–**
–	–	GA+AA	GA+AA	13	36	5.63	2.60–12.16	**<0.001**
***GSTT1***, ***GSTM1*** **and** ***TNFα*****−308G>A**								
Present	Present	GG	–	51	10	R	–	**–**
Present	Present	GA+AA	–	14	36	13.11	5.24–32.8	**<0.001**
Present	Null	GG	–	10	10	5.10	1.68–15.4	**0.002**
Present	Null	GA+AA	–	8	12	7.65	2.49–23.50	**<0.001**
Null	Present	GG	–	5	4	4.08	0.92–17.91	**0.049**
Null	Null	GG	–	7	10	7.28	2.23–23.71	**<0.001**
***GSTT1***, ***GSTM1**,* **and** ***TNFα*****−238G>A**								
Present	Present	–	GG	51	22	R	–	–
Present	Present	–	GA+AA	14	24	3.97	1.73–9.08	**<0.001**
Present	Null	–	GG	11	14	2.95	1.15–7.51	**0.02**
Null	Present	–	GG	6	10	3.86	1.24–11.94	**0.014**
Null	Null	–	GG	7	14	4.63	1.64–13.06	**0.002**

*R-reference. The bold values represent significant values*.

A univariate Cox analysis revealed that patients at stage III according to ISS or those who did not receive auto-HSCT had a 2.92 (*p* = 0.002) and 5.0 (*p* < 0.001) times increased risk of death, respectively (Table 6). Similar findings were observed in the case of disease relapse or progression in MM patients at stage III (HR = 2.62, *p* < 0.001) and without auto-HSCT (HR = 2.66, *p* = 0.001) (Table 6). The multivariate regression analysis also confirmed, that patients without auto-HSCT had a 5.07 times increased risk of death (Table 7). Patients, who did not receive auto-HSCT had 2.13 more chances of disease relapse or progression (Table 7). In contrast, MM patients at stage III or with *GSTM1* present genotype had lower chances (HR = 0.44, *p* = 0.009 or HR = 0.46, *p* = 0.029, respectively) of disease relapse or progression (Table 7).

**Table 6:** Univariate Cox analysis in survival of MM patients.

**Variable**	**Univariate Cox analysis for OS**			**Univariate Cox analysis for PFS**		
	***p*-value**	**HR**	**95% CI**	***p*-value**	**HR**	**95% CI**
***ISS***						
I+II	–	R	–	–	R	–
III	**0.002**	2.92	1.44–5.92	**<0.001**	2.62	1.53–4.49
***Auto-HSCT***						
Yes	–	R	–	–	R	–
No	**<0.001**	5.0	2.02–12.40	**0.001**	2.66	1.45–4.86
***TNFα−308G>A***						
GG	0.53	1.27	0.59–2.75	0.37	1.32	0.71–2.44
GA+AA	–	R	–	–	R	–
***TNFα−238G>A***						
GG	0.97	0.98	0.46–2.07	0.71	0.89	0.49–1.61
GA+AA	–	R	–	–	R	–
***GSTT1***						
Present	0.70	1.18	0.48–2.88	0.99	1.0	0.50–2.01
Null	–	R	–	–	R	–
***GSTM1***						
Present	0.07	2.21	0.9–5.37	0.07	1.77	0.93–3.36
Null	–	R	–	–	R	–
***TNFα−308 and−238***						
GG+GA+GG+GA	0.66	1.24	0.46–3.35	0.57	1.27	0.55–2.93
AA+AA	–	R	–	–	R	–
***TNFα−308 and*****GSTT1****						
GG+present	0.12	0.43	0.14–1.25	0.16	0.47	0.16–1.36
GA+AA+null	–	R	–	–	R	–
***TNFα−308 and*****GSTM1****						
GG+present	0.31	1.49	0.67–3.31	0.24	1.46	0.76–2.80
GA+AA+null	–	R	–	–	R	–
***TNFα−238 and*****GSTT1****						
GG+present	0.58	0.66	0.15–2.86	0.13	0.39	0.11–1.33
GA+AA+null	–	R	–	–	R	–
***TNFα−238 and*****GSTM1****						
GG+present	0.14	4.56	0.60–34.4	0.33	1.60	0.60–4.24
GA+AA+null	–	R	–	–	R	–

*R-reference. The bold values represent significant values*.

**Table 7:** Multivariate Cox analysis in survival of MM patients.

**Variable**	**Multivariate Cox analysis for OS**			**Multivariate Cox analysis for PFS**		
	***p*-value**	**HR**	**95% CI**	***p*-value**	**HR**	**95% CI**
***ISS***						
I+II	–	Reference	–	–	Reference	–
III	0.058	2.22	0.97–5.10	**0.009**	0.44	0.24–0.82
**Auto-HSCT**						
Yes	–	Reference	–	–	Reference	–
No	**0.001**	5.07	1.88–13.68	**0.025**	2.13	1.09–4.15
***TNFα*****−308G>A**						
GG	0.35	1.46	0.65–3.30	0.28	1.44	0.74–2.81
GA+AA	–	Reference	–	–	Reference	–
***TNFα*****−238G>A**						
GG	0.13	0.51	0.22–1.22	0.10	0.58	0.30–1.12
GA+AA	–	Reference	–	–	Reference	–
***GSTT1***						
Present	0.51	1.39	0.51–3.81	0.92	0.96	0.45–2.05
Null	–	Reference	–	–	Reference	–
***GSTM1***						
Present	0.050	2.55	0.99–6.54	**0.029**	0.46	0.23–0.92
Null	–	Reference	–	–	Reference	–

*The bold values represent significant values*.

The analysis of response rate in MM showed that patients at stage III or without auto-HSCT had an increased chance of progressive disease (PD) (Table 8). We did not observe the association of studied polymorphisms with response rate of MM patients (Table 8).

**Table 8:** *TNF-*α and *GSTs* polymorphisms in response rate of MM patients.

**Variable**	**Response rate**	
	**CR+VGPR+PR+SD**	**PD**
	***p*-value**	**OR (95% CI)**
**ISS**		
I+II	–	Reference
III	**<0.001**	5.44 (2.14–13.84)
**Auto-HSCT**		
Yes	–	Reference
No	**0.015**	3.36 (1.22–9.25)
***TNFα*****−308G>A**		
GG	1.0	1.02 (0.39–2.69)
GA+AA	–	Reference
***TNFα*****−238G>A**		
GG	0.45	0.69 (0.26–1.80)
GA+AA	–	Reference
***GSTT1***		
Present	0.60	0.74 (0.24–2.24)
Null	–	Reference
***GSTM1***		
Present	0.30	1.71 (0.61–4.80)
Null	–	Reference
***TNFα*****−308 and−238**		
GG+GG	0.76	0.66 (0.19–2.32)
GA+AA+GA+AA	–	Reference
***TNFα*****−308 and** ***GSTT1***		
GG+present	0.41	0.30 (0.04–1.95)
GA+AA+null	–	Reference
***TNFα*****−308 and** ***GSTM1***		
GG+present	0.90	1.19 (0.27–5.16)
GA+AA+null	–	Reference
***TNFα*****−238 and** ***GSTT1***		
GG+present	0.93	0.56 (0.08–3.69)
GA+AA+null	–	Reference
***TNFα*****−238 and** ***GSTM1***		
GG+present	0.50	3.12 (0.36–27.0)
GA+AA+null	–	Reference

*CR, complete response; VGPR, very good partial response; PR, partial response; SD, stable disease; PD, progressive disease patients, according to response criteria for MM (24, 25). The bold values represent significant values*.

Furthermore, we analyzed potential relationships between clinical and laboratory results and selected genotypes. The −308/−238 GA+AA genotypes were associated with development of MM at an earlier age in comparison to −308/−238 GG−61.14 vs. 66.86 years (*p* = 0.009) and 61.72 vs. 66.52 years (*p* = 0.035), respectively (Table 9). Moreover, higher rates of plasma cells were observed in −308 GG than in GA+AA genotypes (33.2 vs. 23.7%, *p* = 0.038). In MM patients with −238 GG, we observed higher concentration of β2-microglobulin in comparison to −238 GA+AA genotype carriers (6.49 vs. 4.30, *p* = 0.02). In the case of other analyzes, we did not confirm any significant correlations among studied polymorphisms regarding such parameters as free light chain ratio, baseline hemoglobin concentration, levels of albumin, calcium, creatinine, C-reactive protein and estimated glomerular filtration rate (Table 9). Odds ratio was used to analyze the relationship of polymorphisms with chromosomal aberrations in MM patients. The results were statistically insignificant - *GSTT1* null (*p* = 0.19), *GSTM1* null (*p* = 0.20), −308 GA+AA (*p* = 0.80), and −238 GA+AA (*p* = 1.0).

**Table 9:** The clinical values of MM patients included to the study taking into account studied polymorphisms.

**Variables**	**MM patients**	***GSTT1***			***GSTM1***			***TNF−308***			***TNF−238***		
		**Null**	**Present**	**p-value**	**Null**	**Present**	***p*-value**	**GG**	**GA+AA**	***p*-value**	**GG**	**GA+AA**	***p*-value**
Mean age (years)^*^	65.32	65.94	65.19	0.77	65.74	65.16	0.79	66.86	61.14	**0.009**	66.52	61.72	**0.035**
Free light chain ratio^*^	303	506.2	263.3	0.26	284.8	357.0	0.69	272.7	391.7	0.51	369.1	103.5	0.15
% of plasma cells in bone marrow^*^	30.75	27.12	31.46	0.43	28.11	31.76	0.42	33.29	23.73	**0.038**	31.85	27.37	0.34
Albumins (g/dL)^*^	3.57	3.75	3.54	0.23	3.78	3.50	0.059	3.59	3.53	0.72	3.56	3.62	0.69
β2-microglobulin^*^ (mg/L)	5.98	5.62	6.05	0.69	5.85	6.03	0.84	6.16	5.49	0.47	6.49	4.30	**0.02**
Calcium^*^ (mM/L)	2.45	2.41	2.45	0.67	2.46	2.44	0.78	2.47	2.38	0.21	2.45	2.42	0.69
Hemoglobin^*^ (g/dL)	10.38	10.17	10.43	0.60	10.51	10.34	0.68	10.44	10.23	0.63	10.30	10.63	0.45
Creatinine^*^ (mg/dL)	1.57	1.25	1.64	0.38	1.47	1.62	0.69	1.64	1.39	0.53	1.72	1.10	0.12
C-reactive protein^*^ (mg/L)	15.68	10.40	16.79	0.50	13.69	16.46	0.73	13.96	19.81	0.46	12.15	25.68	0.10
Estimated glomerular filtration rate^*^ (mL/min/1.73 m^2^)		63.33	61.18	0.80	63.90	60.64	0.65	60.33	64.92	0.53	60.17	66.51	0.42
PFS (months)	18.32	21.88	17.59	0.37	26.81	15.17	**0.003**	16.83	22.33	0.18	17.41	21.04	0.38
OS (months)	31	32.23	24.77	0.28	34.81	22.79	**0.039**	24.13	31.18	0.23	24.10	31.84	0.20

* *at diagnosis. The bold values represent significant values*.

**Figure 3:**
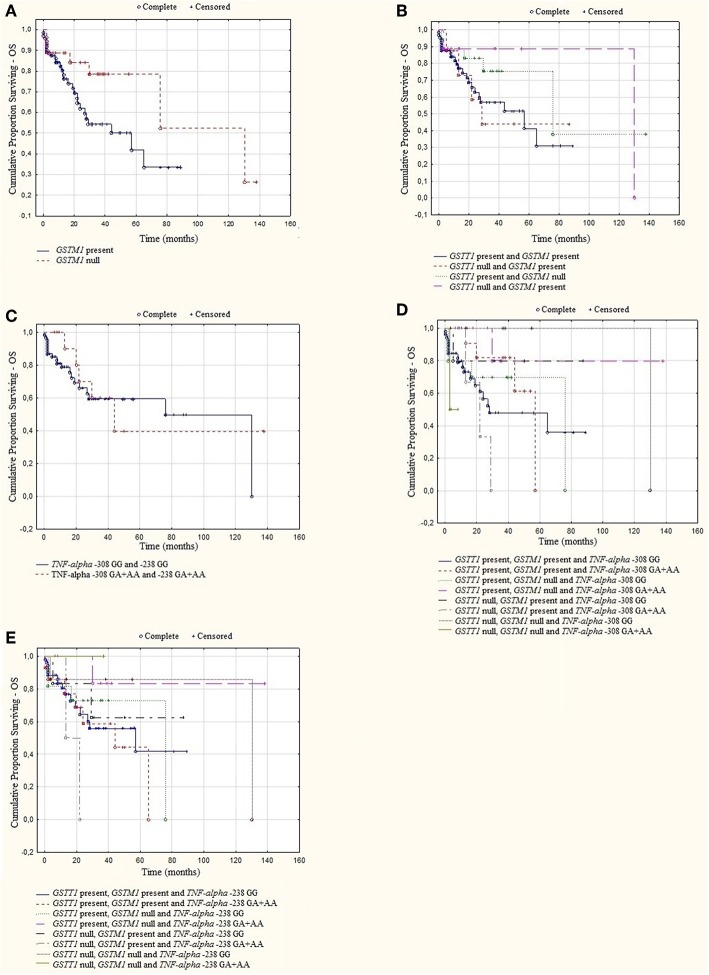
Kaplan-Meier analysis of OS in group of MM patients with **(A)** *GSTM1* genotypes, log-rank test *p* = 0.052; **(B)** *GSTT1* and *GSTM1* genotypes, log-rank test *p* = 0.393; **(C)** *TNF* genotypes, log-rank test *p* = 0.794; **(D)** *GSTT1, GSTM1*, and *TNF-*α−308 genotypes, log-rank test *p* = 0.430; **(E)** *GSTT1, GSTM1*, and *TNF-*α −238 genotypes, log-rank test *p* = 0.659.

**Figure 4:**
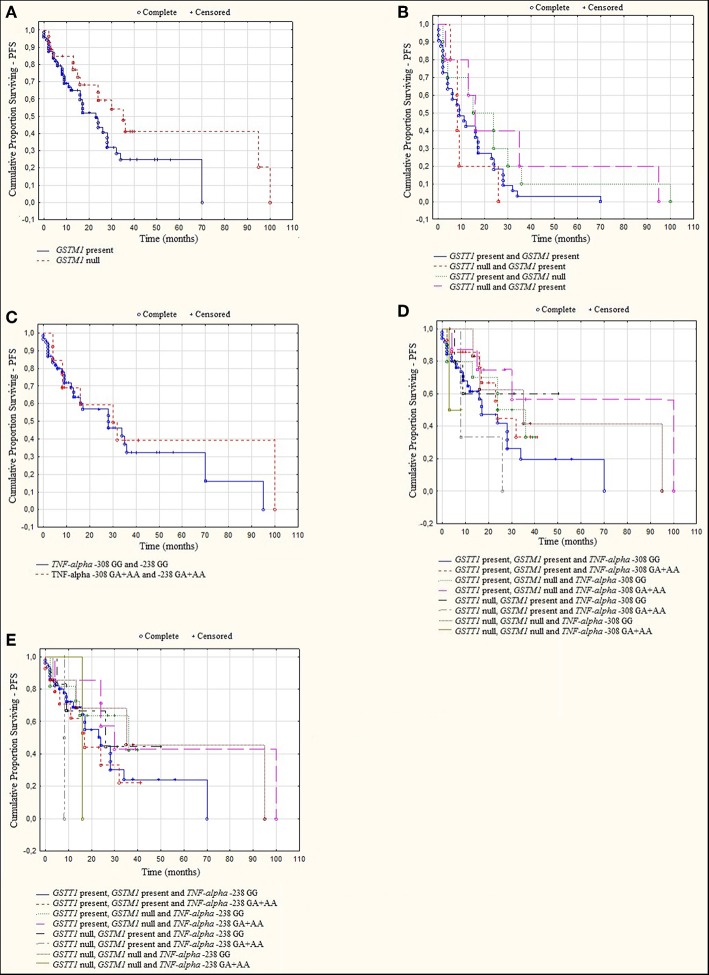
Kaplan-Meier analysis of PFS in group of MM patients with **(A)** *GSTM1* genotypes, log-rank test *p* = 0.056; **(B)** *GSTT1* and *GSTM1* genotypes, log-rank test *p* = 0.501; **(C)** *TNF* genotypes, log-rank test *p* = 0.284; **(D)** *GSTT1, GSTM1*, and *TNF-*α −308 genotypes, log-rank test *p* = 0.102; **(E)** *GSTT1, GSTM1*, and *TNF-*α−238 genotypes, log-rank test *p* = 0.655.

A log rank analysis was performed and did not show any statistical associations between OS, as well as PFS for studied genotypes and the types of treatment (thalidomide vs. bortezomib vs. both—thalidomide and bortezomib). In the log rank test was observed the tendency for shorter OS and PFS in patients with *GSTM1* present genotype (Figures 3, 4). Furthermore, we analyzed (by ANOVA) the association between the studied genotypes and survival of MM patients. Without taking into account the treatment, we found statistically shorter OS and PFS in patients with *GSTM1* present in comparison to those with *GSTM1* null genotype (Table 9).

In *in vitro* studies, bortezomib increased the number of apoptotic and necrotic cells in all studied genotypes. The only statistically significant differences were observed in the number of apoptotic cells at concentration of 12 nM between the genotypes as follows—*TNF-*α −308 GA+AA vs. *GSTT1* present (39.24 vs. 47.46%, respectively), *p* = 0.026; *TNF-*α −308 GA+AA vs. *GSTM1* null (39.24 vs. 49.57%, respectively), *p* = 0.020; *TNF-*α −308 GA+AA vs. *GSTM1* present (39.24 vs. 46.82%, respectively), *p* = 0.046; *TNF-*α −308 GA+AA vs. *TNF-*α −308 GG (39.24 vs. 51.15%, respectively), *p* = 0.001; *TNF-*α −308 GA+AA vs. *TNF-*α −238 GG (39.24 vs. 50.41%, respectively), *p* = 0.004; *TNF-*α −308 GA+AA vs. *TNF-*α −238 GA+AA (39.24 vs. 42.71%, respectively), *p* = 0.017 and *TNF-*α −238 GG vs. *TNF-*α −238 GA+AA (50.41 vs. 42.71%, respectively), *p* = 0.032 (Supplementary Table 3). The lowest number of apoptotic cells was noted at concentration of 12 nM in the *TNF-*α −308 GA+AA genotype.

## Discussion

Genetic polymorphisms in the carcinogen-metabolizing genes are very common and may be associated with the risk of developing different cancers, including hematological malignancies. Among these genes, *GSTs* play a critical role in protecting DNA and cell structures against the ROS (6). Moreover, deletion polymorphisms affect GST activities by detoxifying the metabolites of chemotherapeutic agents and may also affect the treatment of MM (32). Increased production of ROS leads to the accumulation of DNA mutations and activates a positive feedback loop via TNF-α, which promotes the synthesis of inflammatory cytokines. Both the ROS production and activation of TNF-α signaling pathway cause malignant transformation (10, 33). TNF-α is a proinflammatory cytokine that is involved in the stimulation of plasma cell growth and the pathogenesis of MM (34). It is one of particular relevance to MM, where it acts as a growth and survival factor for myeloma cells (35). The functional SNPs in the *TNF-*α gene affect its expression. This may be associated with malignant development and the aggressive course of the disease. The oncogenic transformation of plasma cells stimulates transcription and translation, which results in increased synthesis of immunoglobulins, β2-microglobulin and exacerbation of oxidative stress (36) Malignant cells produce higher amounts of ROS in comparison to normal cells as a result of oncogene activation and/or increased metabolic activity (37, 38). Higher ROS levels may promote cell proliferation and metastasis (39). Considering the above, we hypothesize that polymorphisms of *GSTs* and *TNF-*α genes, individually or combined, may increase the susceptibility to MM and influence the response to treatment of MM patients.

To our knowledge, this is the first study elucidating the correlation of *GSTT1, GSTM1*, and *TNF-*polymorphisms with the risk and the outcome of MM, as well as with response to bortezomib under *in vitro* conditions. Our findings suggest that *GSTT1* null or *TNF-*α −238 GA+AA genotypes are significantly associated with susceptibility to MM. Similar findings were observed in both null genotypes of *GSTT1* and *GSTM1*, as well as in GA+AA genotypes of *TNF-*α −308 and −238 SNPs.

The AA genotypes of *TNF-*α −308 G>A and −238 G>A are associated with increased constitutive and inducible expression of TNF-α (11, 21, 22, 34). The studies of −308 and −238 SNPs of the *TNF-*α gene in MM have shown controversial results (40–42). Davies et al. found that AA genotypes of *TNF-*α −308 SNP increased the risk of MM (43). In contrast, Morgan et al. in 181 MM patients revealed that A variant at position −308 of the *TNF-*α reduced risk of MM (41). In the case of *TNF-*α −238 G>A, some studies showed no association of this polymorphism with MM susceptibility (40–42, 44). Although Li et al. in a meta-analysis described lack of −308 and −238 SNPs association with MM risk in both the overall population and sub-group analysis, our results in Polish, Caucasian MM patients showed a significant correlation of *TNF-*α −238 GA+AA alone or −238 GG in most combinations with higher risk of MM development (45). This may be due to higher *TNF-*α gene expression due to the role of A alleles (increasing transcription rate) or as a result of linkage disequilibrium of G alleles with other functional SNPs involved in the development of MM (40). However, in our study the number of patients with AA genotypes was very low and they were analyzed with heterozygotes, which does not thoroughly show the role of the A allele.

A meta-analysis of *TNF-*α promoter polymorphisms association with MM risk validated a relationship of −308G>A and −238G>A with MM susceptibility, but not with survival and response to bortezomib/thalidomide treatment of MM patients (45). These features were analyzed in our research. However, we did not observe effects of *TNF-*α −308 GG/GA+AA or −238 GG/GA+AA genotypes on treatment with bortezomib and/or thalidomide in MM patients. This finding is consistent with results obtained by Du et al. in 210 MM patients (40). Moreover, they revealed that patients with *TNF-*α −238 GG genotypes have significantly longer OS (40). We found that *TNF-*α −308 GA+AA and −238 GA+AA genotypes were associated with earlier onset of MM development, which may be caused by higher expression of TNF-α. However, these genotypes did not affect OS and PFS. In *TNF-*α −308 GG and −238 GG genotype analysis, we observed a statistical significant relationship with higher levels of plasma cells and β2-microglobulin, respectively. These clinical values are negative prognostic factors for MM patients. In our research, −308/−238 GG genotypes increased the MM risk in combination with the null genotypes *GSTT1* and/or *GSTM1*. It is possible that genotypes of *TNF-*α exert only a minor effect and are linked to other SNPs.

In the case of *GST* genotypes and their impact on the MM risk, our results are compatible with other studies in hematological malignancies including MM (18, 46, 47). For example, Chen and co-authors described that *GSTM1* null genotype combined with *CYP1A1* and *CYP2D6* heterozygous mutant genotypes were correlated with an elevated risk of acute non-lymphoblastic leukemia (ANLL) (8). Yuille and co-workers studied the relationship between genotypes of *GSTM1, GSTT1, GSTP1*, and chronic lymphocytic leukemia (CLL) (48). They found that the risk of CLL was increased with the *GSTM1* null and *GSTT1* null genotypes (48). In our study individuals with the *GSTT1* null or with both the *GSTT1* null and *GSTM1* null genotypes showed a higher risk of MM compared to patients carrying non-deleted *GSTT1*/*GSTM1* genotypes. Moreover, the *GSTM1* null genotype was alone associated with increased risk of MM development (at the level of tendency). Deletion of GSTs alleles resulted in lack of enzyme activity. This may increase the level of carcinogens and mutation rates in genes regulating cell cycle progression, which result in higher cell proliferation rates, including proliferation of plasma cells.

Both MM and CLL are derived from B cells, which points to the role of *GSTT1* and *GSTM1* polymorphisms in the pathogenesis of B-cell malignancies. Few studies are focused on *GSTT1* and *GSTM1* polymorphisms in MM (18, 19). In our research, we observed the association of the *GSTM1* present genotype with shorter OS (at the level of tendency) and PFS of MM patients. Multivariate Cox analysis showed that patients with *GSTM1* present genotype had lower chances for longer PFS, which is consistent with the research of Lopes-Aguiar et al. (19). They noted shorter OS and event-free survival (EFS) in patients with *GSTM1* present genotype who did not receive ASCT after chemotherapy (19). It is possible that the presence of the *GSTM1* gene and activity of the respective GST enzyme affects the treatment of MM patients. GSTs are involved in the elimination of chemical carcinogens and may neutralize the effect of bortezomib and thalidomide.

In this paper we analyzed, for the first time, the *GSTT1, GSTM1*, and *TNF-*α SNPs in the survival and response to therapy of MM patients. *TNF-*α −308 GG/GA+AA or −238 GG in combination with *GSTT1* and *GSTM1* polymorphisms increased the risk of MM. Interestingly, the presence of −238 GA+AA with the null genotypes of *GSTT1* and/or *GSTM1* did not influence disease risk. It may indicate a possible protective role of the −238 GA+AA genotype. These combined associations have not been previously reported.

Bortezomib causes an accumulation of unfolded proteins, followed by endoplasmatic reticulum stress and cell death via multiple pathways, including overproduction of ROS (49). ROS generation precedes the initiation of bortezomib-induced apoptosis (16). Taking this into account, we have evaluated the relationship between studied polymorphisms and the response to bortezomib under *in vitro* conditions. This association was not previously studied. We hypothesized that *GSTT1, GSTM1*, and *TNF-*α polymorphisms may interact with the response to bortezomib treatment. We observed statistically significant differences between studied polymorphisms in the number of apoptotic cells at 12 nM concentration of bortezomib. The lowest number of apoptotic cells was found in the *TNF-*α −308 GA+AA genotype. In the human study, higher levels of plasma cells were observed in MM patients with the −308 GG, not with the −308 GA+AA genotype. Moreover, low number of apoptotic cells was found in −238 GA+AA, which was associated alone and in most combinations with a higher risk of MM development. The inconsistent results of the *in vitro* and human study may be due to a low sample size. Furthermore, we found spontaneous apoptosis and necrosis in cell cultures without bortezomib, which may be due to laboratory conditions. Alternatively, myeloma cell lines could be used. However, in the case of studied polymorphisms, reference cell lines with all studied genotypes are not available, which may be used in the presented *in vitro* study.

A limitation of our study is the relatively small sample size, in part due to the low incidence of the disease. Further analysis on a larger cohort can help better understand the significance of *GSTT1, GSTM1, TNF-*α −308 G>A and −238 G>A polymorphisms in the pathobiology of MM, especially in the case of disease outcome.

In conclusion, these data underscore the relationship of *TNF-*α −238 GA+AA and *GSTT1* null genotypes with increased risk of MM. *TNF-*α −308 genotypes in combinations with *GSTT1/GSTM1* genotypes were associated with increased susceptibility to MM. The *TNF-*α −238 GG was associated with higher MM risk, but only in the absence of the *GSTT1* and/or *GSTM1* genotypes. The *GSTM1* genotypes affected the outcome of MM patients. However, we found no association of the studied polymorphisms with the response rate and treatment of thalidomide and/or bortezomib. Under *in vitro* conditions, bortezomib significantly increased the number of apoptotic cells at 12 nM in *GSTT1* present, *TNF-*α −308 GG and all genotypes of *GSTM1* and *TNF-*α −238. Further analysis on a larger cohort is necessary to better understand the influence of *GSTT1, GSTM1*, and *TNF-*α polymorphisms on the MM development and progression.

## Data Availability Statement

This manuscript contains previously unpublished data. The name of the repository and accession number are not available.

## Ethics Statement

The positive opinion was obtained from the Bioethics Committee (no. KE-0254/165/2013 and no. KE-0254/337/2016), according with the ethical standards established by Helsinki Declaration.

## Author Contributions

SZ, SP-M, MW-L, SC, and IK-P carried out the experiment. SZ, SP-M, MW-L, and IK-P performed the molecular analysis of GSTs and TNF-alpha polymorphisms. SC performed cytogenetic analysis. SZ and SP-M performed *in vitro* study with bortezomib. MH and AF were involved in planning and supervised the work. SZ, AS-S, and WS processed the experimental data and performed the analysis. SZ, SP-M, and WS designed the figures. SZ, AS-S, and WS wrote the manuscript with support from MH and AF. All authors discussed the results and commented on the manuscript.

### Conflict of Interest

The authors declare that the research was conducted in the absence of any commercial or financial relationships that could be construed as a potential conflict of interest.
