# Supplementary material for: The Association of GSTT1, GSTM1, and TNF-α Polymorphisms With the Risk and Outcome in Multiple Myeloma
Source: Front Oncol. 2019 Oct 11;9:1056. doi: 10.3389/fonc.2019.01056 (PMC6798955; doi:10.3389/fonc.2019.01056)
Supplement: Supplementary file 1 [file Data_Sheet_1.docx]

**Supplementary material**

**Table 1. The characteristics at diagnosis of MM patients included to the study.**

| Variables | MM patients  n=100 |
| --- | --- |
| Sex | |
| Male | 53 |
| Female | 47 |
| Age* | |
| Mean age (years) | 65.32 |
| Type of MM* | |
| IgG | 56 |
| IgA | 25 |
| Light chain | 19 |
| Free light chain ratio | 303 |
| Stage according to the International Staging System* | |
| I | 28 |
| II | 30 |
| III | 42 |
| Renal failure* | |
| No | 82 |
| Yes | 18 |
| The stage of kidney disease* | |
| G1 | 30 |
| G2 | 28 |
| G3A | 16 |
| G3B | 12 |
| G4 | 7 |
| G5 | 7 |
| Plasma cells* | |
| Percentage of plasma cells in bone marrow, M± SD | 30.75  ±20.42 |
| Cytogenetic changes* | |
| del(17p13.1) | 14 |
| t(4;14) | 15 |
| t(14;16) | 2 |
| Clinical values* | |
| Albumins (g/dL)  M± SD | 3.57  ±0.66 |
| β2-microglobulin (mg/L),  M± SD | 5.98  ±4.02 |
| Calcium (mM/L),  M± SD | 2.45  ±0.32 |
| Hemoglobin (g/dL),  M± SD | 10.38  ±1.87 |
| Creatinine (mg/dL),  M± SD | 1.57  ±1.67 |
| C-reactive protein (mg/L),  M± SD | 15.68  ±33.48 |
| Chemotherapy | |
| Cyclophosphamide, Thalidomide, Dexamethasone (CTD) | 49 |
| Velcade, Cyclophosphamide, Dexamethasone (VCD) | 29 |
| Velcade, Thalidomide, Dexamethasone (VTD) | 22 |
| Survival | |
| Progression free survival (months), M± SD | 18.32  ±18.01 |
| Overall survival (months)  M± SD | 26.04  ±25.93 |

*at diagnosis

M-mean, SD – standard deviation.

**Table 2. Inclusion and exclusion criteria for MM patients and blood donors.**

|  | **Inclusion criteria** | **Exclusion criteria** |
| --- | --- | --- |
| MM patients | -Newly diagnosed MM patients who require start active treatment according to the IMWG published in 2014.  -Signed informed consent  -Patients must have measurable disease, defined as follows:   - For secretory MM, measurable disease is defined as the presence of quantifiable monoclonal component, ≥ 0.5 g/dL - For poor secretory or non-secretory MM, the level of the affected serum free light chain must be ≥ 10 mg/dL (≥ 100 mg/L, with an abnormal free light-chain ratio)   -Eastern Cooperative Oncology Group (ECOG) Performance status ≤3  -Life expectancy more than 3 months | -Active smoldering MM.  -Active plasma cell leukemia.  -Documented systemic amyloid light chain amyloidosis.  -Active central nervous system involvement with MM.  -Active other hematologic malignancy or solid tumor. |
| Control group | -Age: 18 age and older  -Signed informed consent  -Agree to have blood donated and stored for research. | -Known to be infected with HIV, syphilis, tuberculosis, hepatitis B or hepatitis C.  -A condition in which repeated blood draws or injections pose more than minimal risk for the subject such as hemophilia, other severe coagulation disorders or significantly impaired venous access.  -A condition that requires active medical intervention or monitoring to avert serious danger to the participant's health or well-being. |

**Table 3. The effect of different bortezomib doses on bone marrow cells apoptosis, viability and necrosis** (average values).

| Genotypes | Bortezomib/DMSO | | | | | |
| --- | --- | --- | --- | --- | --- | --- |
|  | Control  0,1% DMSO  (0nM) | 1nM | 2nM | 4nM | 8nM | 12nM |
| Apoptotic cells (%) | | | | | | |
| Mean | 5.45 | 15.44 | 17.49 | 25.55 | 37.52 | 47.68 |
| *GSTT1* null | 8.64 | 15.86 | 14.13 | 22.50 | 28.84 | 48.94 |
| *GSTT1* present | 4.91 | 15.37 | 18.07 | 26.07 | 39.0 | 47.46 |
| *GSTM1* null | 7.27 | 18.29 | 16.09 | 25.45 | 37.51 | 49.57 |
| *GSTM1* present | 4.62 | 14.15 | 18.13 | 25.59 | 37.52 | 46.82 |
| *TNFα* -308 GG | 5.09 | 16.40 | 17.47 | 26.53 | 39.71 | 51.15 |
| *TNFα* -308 GA+AA | 6.32 | 13.11 | 17.55 | 23.17 | 32.20 | 39.24 |
| *TNFα* -238 GG | 6.21 | 15.55 | 16.96 | 26.22 | 37.93 | 50.41 |
| *TNFα* -238 GA+AA | 4.07 | 15.24 | 18.45 | 24.31 | 36.77 | 42.71 |
| Necrotic cells (%) | | | | | | |
| Mean | 2.43 | 4.35 | 6.65 | 11.69 | 12.84 | 14.93 |
| *GSTT1* null | 1.81 | 3.37 | 4.72 | 8.26 | 8.93 | 10.81 |
| *GSTT1* present | 2.54 | 4.52 | 6.98 | 12.28 | 13.50 | 15.63 |
| *GSTM1* null | 3.80 | 4.71 | 7.45 | 10.73 | 13.09 | 13.25 |
| *GSTM1* present | 1.81 | 4.19 | 6.28 | 12.13 | 12.72 | 15.70 |
| *TNFα* -308 GG | 2.42 | 4.44 | 6.27 | 12.48 | 13.43 | 15.04 |
| *TNFα* -308 GA+AA | 2.47 | 4.13 | 7.56 | 9.79 | 11.39 | 14.67 |
| *TNFα* -238 GG | 2.08 | 4.11 | 5.84 | 10.66 | 10.86 | 13.59 |
| *TNFα* -238 GA+AA | 3.07 | 4.80 | 8.11 | 13.58 | 16.44 | 17.38 |
| Viable cells (%) | | | | | | |
| Mean | 89.17 | 79.58 | 76.08 | 66.18 | 59.71 | 53.32 |
| *GSTT1* null | 90.21 | 82.43 | 81.21 | 70.55 | 65.04 | 61.67 |
| *GSTT1* present | 89.0 | 79.10 | 75.20 | 65.43 | 58.80 | 51.90 |
| *GSTM1* null | 84.10 | 76.54 | 76.45 | 66.72 | 62.32 | 53.31 |
| *GSTM1* present | 91.48 | 80.97 | 75.91 | 65.93 | 58.53 | 53.32 |
| *TNFα* -308 GG | 89.37 | 79.60 | 76.41 | 64.99 | 59.64 | 53.85 |
| *TNFα* -308 GA+AA | 88.70 | 79.53 | 75.28 | 69.07 | 59.90 | 52.04 |
| *TNFα* -238 GG | 88.47 | 79.25 | 77.0 | 65.96 | 60.69 | 53.55 |
| *TNFα* -238 GA+AA | 90.46 | 80.19 | 74.40 | 66.57 | 57.92 | 52.89 |
| p-values | 0.07 | 0.95 | 0.94 | 0.93 | 0.78 | 0.33 |
